# A combination Alzheimer’s therapy targeting BACE1 and neprilysin in 5XFAD transgenic mice

**DOI:** 10.1186/s13041-015-0110-5

**Published:** 2015-03-25

**Authors:** Latha Devi, Masuo Ohno

**Affiliations:** Center for Dementia Research, Nathan Kline Institute, 140 Old Orangeburg Road, Orangeburg, NY 10962 USA; Department of Psychiatry, New York University Langone Medical Center, New York, NY 10016 USA

**Keywords:** Alzheimer’s disease, Amyloid-β, β-Secretase, BACE1, Neprilysin, C99, Learning and memory, Fear conditioning, Cholinergic neuron, 5XFAD

## Abstract

**Background:**

Accumulating evidence indicates that partial inhibition of β-site APP-cleaving enzyme 1 (BACE1), which initiates amyloid-β (Aβ) production, mitigates Alzheimer’s disease (AD)-like pathologies and memory deficits in a battery of transgenic mouse models. However, our previous investigations suggest that therapeutic BACE1 suppression may be beneficial only if targeted on earlier stages of AD and encounter dramatic reductions in efficacy during disease progression. This study was designed to test the possibility that a combination approach, aimed at inhibiting BACE1 and boosting neprilysin (a major Aβ-degrading enzyme) activities, may be able to mechanistically overcome the limited efficacy of anti-Aβ therapy in advanced AD.

**Results:**

After crossbreeding between BACE1 heterozygous knockout (BACE1^+/−^), neprilysin transgenic (NEP) and 5XFAD mice, we analyzed the resultant mice at 12 months of age when 5XFAD controls showed robust amyloid-β (Aβ) accumulation and elevation of BACE1 expression (~2 folds). Although haploinsufficiency lowered BACE1 expression by ~50% in concordance with reduction in gene copy number, profound β-amyloidosis, memory deficits and cholinergic neuron death were no longer rescued in BACE1^+/−^ · 5XFAD mice concomitant with their persistently upregulated BACE1 (i.e., equivalent to wild-type control levels). Notably, neprilysin overexpression not only prevented Aβ accumulation but also suppressed the translation initiation factor eIF2α-associated elevation of BACE1 and lowered levels of the β-secretase-cleaved C-terminal fragment of APP (C99) in NEP · 5XFAD mice. Interestingly, these markers for β-amyloidogenesis in BACE1^+/−^ · NEP · 5XFAD mice were further reduced to the levels reflecting a combination of single BACE1 allele ablation and the abolishment of translational BACE1 upregulation. However, since neprilysin overexpression was striking (~8-fold relative to wild-type controls), memory impairments, cholinergic neuronal loss and β-amyloidosis were similarly prevented in NEP · 5XFAD and BACE1^+/−^ · NEP · 5XFAD mice.

**Conclusions:**

Our findings indicate that robust overexpression of neprilysin is sufficient to ameliorate AD-like phenotypes in aged 5XFAD mice. We also found that Aβ-degrading effects of overexpressed neprilysin can block deleterious BACE1-elevating mechanisms that accelerate Aβ production, warranting further study to test whether interventions moderately activating neprilysin may be useful for boosting the limited efficacy of therapeutic BACE1 inhibition in treating AD with established Aβ pathology.

## Background

The β-secretase, called β-site amyloid precursor protein-cleaving enzyme 1 (BACE1), was identified as an aspartyl protease that initiates the production of amyloid-β (Aβ) peptides [1]. Given pathogenic roles of Aβ in AD [2,3], BACE1 is one of the prime therapeutic targets to prevent or treat this devastating neurodegenerative disorder [4-7]. It has been demonstrated that homozygous BACE1 knockout (BACE1^−/−^) prevents the development of AD-like pathologies and memory deficits in different transgenic lines of amyloid precursor protein (APP)-overexpressing mice [8-11]. Furthermore, BACE1 haploinsufficiency (BACE1^+/−^; i.e., a therapeutic relevant model for 50% suppression) [12-18] as well as chronic treatments with bioavailable small-molecule BACE1 inhibitors (e.g., GRL-8234 and TAK-070) [19-21] has been reported to partially reduce cerebral Aβ concentrations and mitigate amyloid plaque and tau pathologies, cholinergic neuron loss, mitochondrial dysfunction, hippocampal synaptic failure, and memory deficits in APP mice. However, some of these studies raise concern that the beneficial outcomes associated with partial BACE1 inhibition may decline during the progression of AD [11,12,15,16,18,21].

It has been demonstrated that Aβ accumulation induces BACE1 elevation in neurons surrounding the amyloid core of plaques, which in turn further accelerates Aβ production and plaque growth in brains of the 5XFAD mouse model and AD patients [22-26]. Meanwhile, previous studies with neprilysin transgenic and knockout mice have clearly shown that neprilysin-dependent degradation of Aβ is critical for suppressing plaque development [27-30]. Notably, we found that partial suppression of BACE1 with haploinsufficiency or the β-secretase inhibitor GRL-8234 was no longer able to exert beneficial effects including memory improvements in advanced stages of 5XFAD mice (≥12 months of age) suffering from robust Aβ deposition, since it failed to reverse BACE1 elevation and neprilysin reduction in the brain [15,18,21]. Therefore, our results suggest that monotherapeutic approaches targeting BACE1 may not be sufficient to block a detrimental feed-forward link between neprilysin reduction, increased levels of Aβ plaque formation and BACE1 elevation during the course of AD progression. In this study, we tested whether a combination strategy that boosts Aβ degradation (neprilysin overexpression) and arrests Aβ production (BACE1 haploinsufficiency) may be useful for mechanistically increasing the limited therapeutic efficacies in 12-month-old 5XFAD mice.

## Results

### Effects of BACE1 haploinsufficiency combined with neprilysin overexpression on β-amyloidogenic processing of APP in aged 5XFAD mice

**Figure 1:**
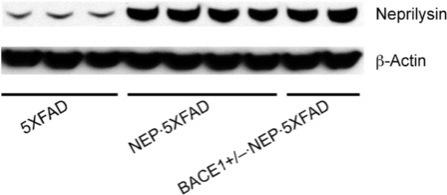
**Western blot analysis of transgenic overexpression of neprilysin in 5XFAD mice.** Equivalent levels of neprilysin are overexpressed in NEP · 5XFAD and BACE1^+/−^ · NEP · 5XFAD mouse brains (~8-fold relative to 5XFAD controls).

We used 5XFAD APP/presenilin-1 (PS1) transgenic mice that represent a rapid-onset and aggressive amyloid model based on a combination of five familial AD (FAD) mutations and the consequent acceleration of neurotoxic Aβ42 production [9,10,31]. 5XFAD mice begin to develop visible Aβ deposition as early as 2 months of age and exhibit memory declines on a battery of hippocampus-dependent tasks around 6 months concomitant with synaptic dysfunction at the Schaffer collateral-CA1 pathway [9,13,31-38]. We tested the effects of heterozygous BACE1 deletion (BACE1^+/−^), neprilysin overexpression (NEP) and their combination (BACE1^+/−^ · NEP) in 5XFAD mice at 12 months of age, which show extensive Aβ plaque pathology concomitant with significant BACE1 elevation and neprilysin reduction and are no longer responsive to rescue by BACE1 haploinsufficiency [15,16,18]. We first compared the degree of neprilysin overexpression by quantitative immunoblot analysis of brain samples (Figure 1). As previously reported in hAPP-J20 transgenic mice engineered to overexpress neprilysin [29,30], we confirmed robust overexpression of neprilysin (859 ± 20%) in NEP · 5XFAD mice relative to 5XFAD controls. Moreover, we found that the addition of BACE1^+/−^ gene ablation to these mice (i.e., BACE1^+/−^ · NEP · 5XFAD) did not affect overexpression levels of neprilysin (864 ± 31%).

**Figure 2:**
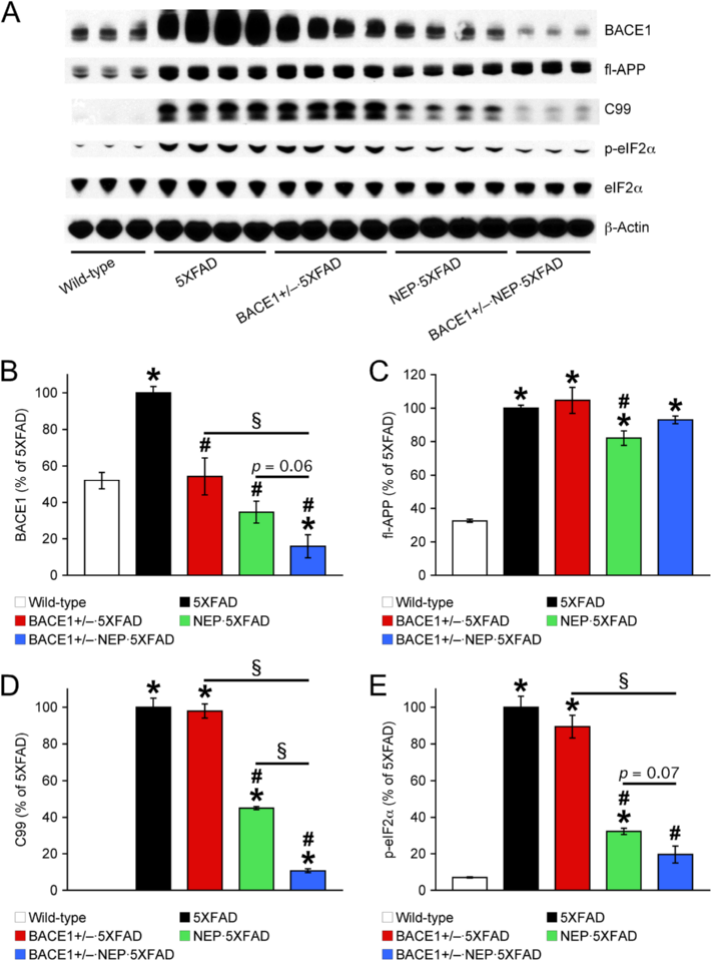
**Effects of a combination of BACE1 haploinsufficiency and neprilysin overexpression on β-amyloidogenic processing of APP in 12-month-old 5XFAD mice. (A)** Representative immunoblots of protein extracts from hemibrain homogenates of mice. **(B–E)** Immunoreactive bands were quantified and expressed as the percentage of 5XFAD control mice (*n* = 3–4 mice per group). Note that BACE1 expression is reduced below wild-type controls in BACE1^+/−^ · NEP · 5XFAD mice, as expected by a single BACE1 allele ablation and the complete abolishment of eIF2α phosphorylation-dependent translational upregulation. Consequently, the β-secretase-cleaved C-terminal fragment of APP (C99) is also dramatically reduced in these mice. * *p* < 0.05 vs. wild-type, ^#^ *p* < 0.05 vs. 5XFAD, ^§^ *p* < 0.05 vs. BACE1^+/−^ · NEP · 5XFAD. All data are presented as mean ± SEM.

Next, we investigated changes in the β-amyloidogenic processing of APP (Figure 2). BACE1 expression levels in 12-month-old 5XFAD mice were significantly increased up to ~2 folds as compared with those in wild-type control mice (*p* < 0.05) (Figure 2A, B). Consistent with our previous results [15,18], although haploinsufficiency reduced BACE1 expression by ~50% in 5XFAD mice (*p* < 0.05), the residual levels of BACE1 in BACE1^+/−^ · 5XFAD mouse brains were equivalent to wild-type control levels (i.e., twice the gene copy number). Interestingly, neprilysin overexpression in 5XFAD mice also reversed the upregulation of BACE1 to wild-type levels (*p* < 0.05). Of note, BACE1 levels were indistinguishable between neprilysin transgenic and wild-type control mice (data not shown), excluding the possibility that neprilysin overexpression by itself may directly affect BACE1 expression. A significant reduction or trend toward reduction of BACE1 was observed in BACE1^+/−^ · NEP · 5XFAD mice as compared to BACE1^+/−^ · 5XFAD (*p* < 0.05) or NEP · 5XFAD (*p* = 0.06) mice, indicating that a combination of BACE1 haploinsufficiency and neprilysin overexpression in 5XFAD mice can further reduce BACE1 expression below wild-type control levels (*p* < 0.05). Meanwhile, overexpression levels of full-length APP were not affected except for the NEP · 5XFAD group, which showed a slight but significant decrease compared with 5XFAD controls (*p* < 0.05) (Figure 2A, C). The β-secretase-cleaved C-terminal fragment of APP (β-CTF or C99) in 5XFAD mice at 12 months of age was not affected by BACE1 haploinsufficiency, whereas C99 levels in NEP · 5XFAD mice were significantly lower than those in 5XFAD controls (*p* < 0.05) (Figure 2A, D). Moreover, in accordance with the robust reduction of BACE1 expression, C99 levels were also further reduced in BACE1^+/−^ · NEP · 5XFAD mice as compared with BACE1^+/−^ · 5XFAD and NEP · 5XFAD mice (*p* < 0.05).

Recent findings from our laboratory and others have demonstrated that translational mechanisms through overactivation of the eukaryotic initiation factor-2α (eIF2α) phosphorylation pathway [15,18,24,39-43] rather than transcriptional mechanisms [22,44,45] account for BACE1 elevation found in advanced stages of 5XFAD mice as well as sporadic AD cases. Therefore, we compared levels of phosphorylated eIF2α (Figure 2A, E) in association with the alterations of BACE1 expression. In agreement with our previous studies [15,18], haploinsufficiency lowered BACE1 expression by ~50% in concordance with the reduction of gene copy number but failed to affect phospho-eIF2α-dependent BACE1-elevating mechanisms in 12-month-old 5XFAD mice; therefore, BACE1 expression in BACE1^+/−^ · 5XFAD mice remained upregulated or equivalent to wild-type levels. Importantly, neprilysin overexpression significantly suppressed eIF2α phosphorylation without affecting total eIF2α levels in 5XFAD mice (*p* < 0.05), leading to the reversal of translational BACE1 upregulation. Moreover, a combination of BACE1 haploinsufficiency and neprilysin overexpression in 5XFAD mice almost completely blocked eIF2α phosphorylation, which in turn was able to lower BACE1 expression below wild-type controls in BACE1^+/−^ · NEP · 5XFAD mice as a consequence of the ablation of a single BACE1 allele and the blockade of translational upregulation.

### Effects of BACE1 haploinsufficiency combined with neprilysin overexpression on β-amyloidosis in aged 5XFAD mice

**Figure 3:**
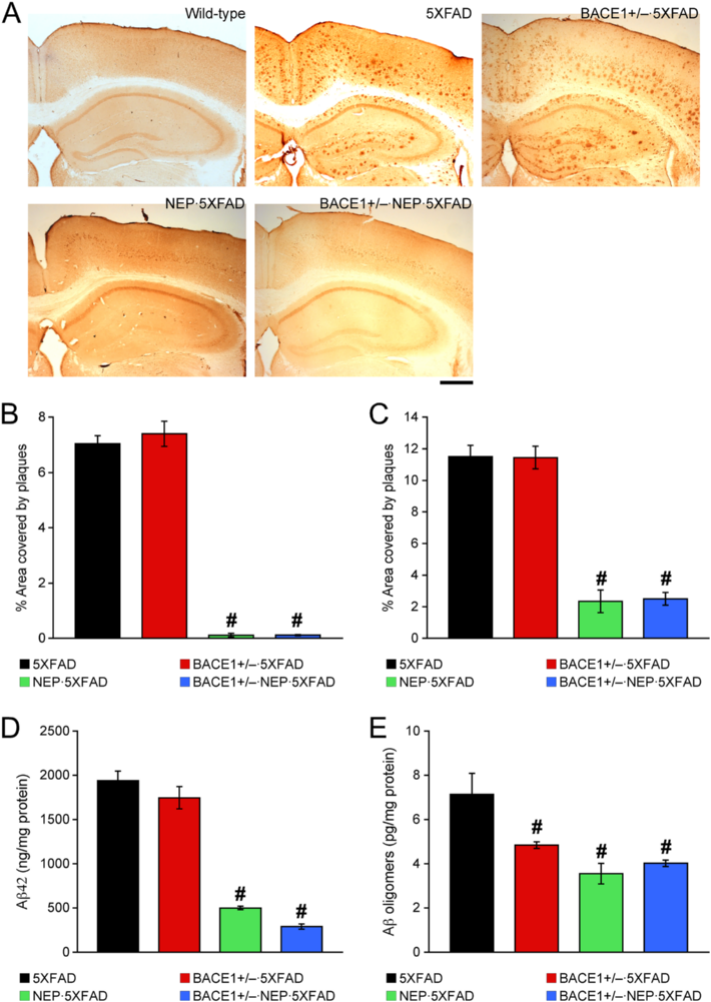
**Effects of a combination of BACE1 haploinsufficiency and neprilysin overexpression on β-amyloidosis in 12-month-old 5XFAD mice. (A)** Brain sections were immunostained with the 6E10 anti-Aβ antibody. Shown are representative photomicrographs of the hippocampal and cortical regions. Scale bar = 500 μm. **(B, C)** Percentage area occupied by Aβ deposits in the hippocampus **(B)** and cerebral cortex **(C)** was measured for quantification (*n* = 2–5 mice per group). **(D, E)** Levels of total Aβ42 in guanidine extracts **(D)** and soluble Aβ oligomers **(E)** were quantified by sandwich ELISAs and expressed in nanograms and picograms per milligram of total protein, respectively (*n* = 4–6 mice per group). Significant and equivalent reductions in all Aβ measurements are observed in NEP · 5XFAD and BACE1^+/−^ · NEP · 5XFAD mice. ^#^ *p* < 0.05 vs. 5XFAD. All data are presented as mean ± SEM.

We further examined how Aβ plaque loads were affected by BACE1 and/or neprilysin manipulations in 5XFAD mice (Figure 3A). In accordance with our previous findings [15,18], we confirmed that BACE1 haploinsufficiency alone was not sufficient to reduce extensive Aβ deposition found in the hippocampus (Figure 3B) or cerebral cortex (Figure 3C) of 5XFAD mice at 12 months of age (*n* = 2). In contrast, as reported in the hAPP-J20 mouse model [29,30], transgenic overexpression of neprilysin almost completely prevented Aβ plaque formation in 5XFAD mice (*p* < 0.05). A combination of BACE1 haploinsufficiency and neprilysin overexpression also similarly blocked plaque development (*p* < 0.05), indicating no further reduction in brain Aβ burden in BACE1^+/−^ · NEP · 5XFAD mice as compared with NEP · 5XFAD mice. Consistent with Aβ immunostaining, sandwich ELISAs also showed that total Aβ42 levels in 5 M guanidine-extracted brains were significantly and equivalently reduced in NEP · 5XFAD and BACE1^+/−^ · NEP · 5XFAD mice (*p* < 0.05), but not in BACE1^+/−^ · 5XFAD mice, as compared with 5XFAD controls (Figure 3D). Interestingly, ELISAs specific to oligomeric forms of Aβ revealed that all of BACE1^+/−^ deletion, neprilysin overexpression and their combination induced similar levels of reductions in soluble Aβ oligomers in 5XFAD mice (*p* < 0.05) (Figure 3E).

### Effects of BACE1 haploinsufficiency combined with neprilysin overexpression on memory deficits and cholinergic neuron loss in aged 5XFAD mice

**Figure 4:**
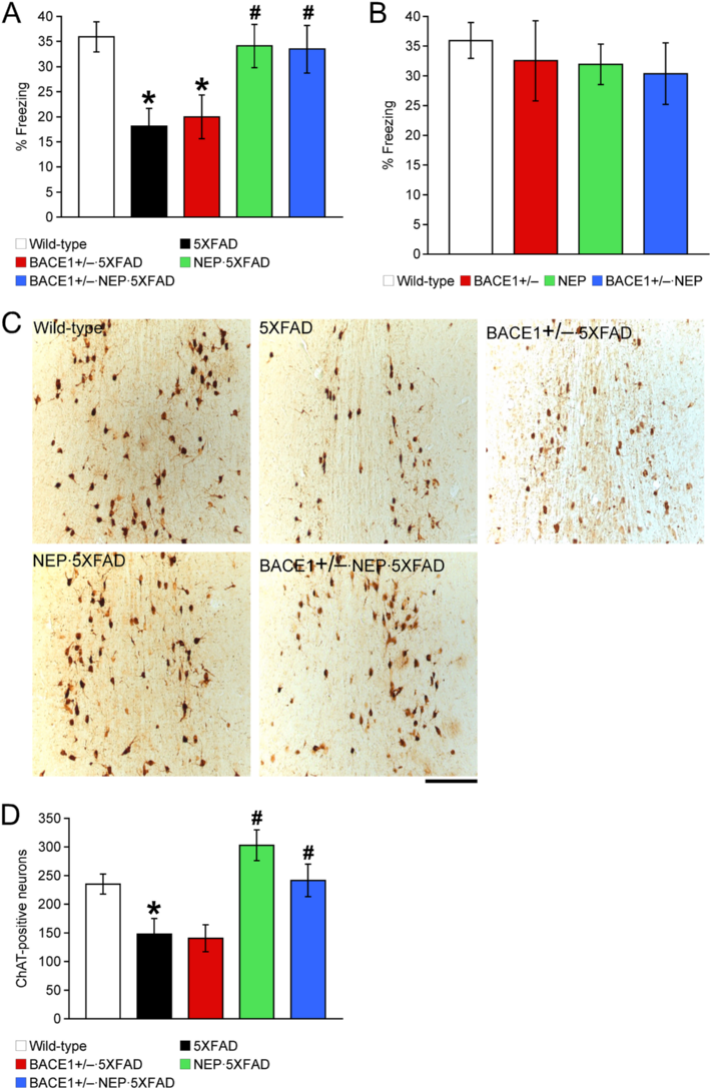
**Effects of a combination of BACE1 haploinsufficiency and neprilysin overexpression on memory deficits and cholinergic neuron loss in 12-month-old 5XFAD mice. (A, B)** Mice were trained with a CS-US pairing for contextual fear conditioning (*n* = 6–11 mice per group). 5XFAD mice showed significantly lower levels of contextual freezing than wild-type mice when tested 24 h after training. NEP · 5XFAD and BACE1^+/−^ · NEP · 5XFAD mice are rescued almost completely back to wild-type control levels of contextual memory. **(C)** Brain sections were immunostained for the cholinergic marker ChAT. Shown are representative photomicrographs of ChAT-immunoreactive neurons in the medial septum of mice. Scale bar = 200 μm. **(D)** The number of ChAT-positive neurons in the medial septum and the vertical limb of the diagonal band (Ch1/2), which provide the cholinergic innervation to the hippocampus, was counted for quantification (*n* = 2–5 mice per group). Cholinergic neuronal death associated with 5XFAD is prevented in NEP · 5XFAD and BACE1^+/−^ · NEP · 5XFAD mice. * *p* < 0.05 vs. wild-type, ^#^ *p* < 0.05 vs. 5XFAD. All data are presented as mean ± SEM.

Finally, we examined whether genetic manipulations of BACE1 and/or neprilysin can rescue memory deficits in 5XFAD mice, using the hippocampus-dependent contextual fear conditioning paradigm (Figure 4A). Wild-type control mice exhibited a robust conditioned fear response as assessed by freezing (the absence of all but respiratory movements) when placed back into the conditioning chamber 24 h after training. 5XFAD mice at 12 months of age showed significantly reduced levels of freezing compared with wild-type controls (*p* < 0.05), whereas contextual memory remained impaired in BACE1^+/−^ · 5XFAD mice as reported previously [18]. In contrast, contextual memory deficits in 5XFAD mice were rescued almost completely back to wild-type levels in NEP · 5XFAD and BACE1^+/−^ · NEP · 5XFAD mice (*p* < 0.05). Meanwhile, freezing levels were indistinguishable between wild-type, BACE1^+/−^, NEP and BACE1^+/−^ · NEP mice (Figure 4B), demonstrating that BACE1^+/−^ reduction, neprilysin overexpression and their combination does not affect baseline memory performances on the non-5XFAD transgenic background.

The septohippocampal cholinergic pathway plays an important role in memory performances including the contextual fear conditioning [46], while cholinergic neuron loss is found in several AD transgenic mouse models such as 5XFAD and APP23 [15,42,47]. Therefore, we further examined whether BACE1 and/or neprilysin manipulations can rescue neurodegeneration by analyzing cholinergic neurons in the medial septum and the vertical limb of the diagonal band (Ch1/2) that provide the cholinergic innervation to the hippocampus (Figure 4C). Immunostaining for choline acetyltransferase (ChAT: a cholinergic marker) revealed that the number of cholinergic neurons was significantly reduced in 12-month-old 5XFAD mice as compared with that of wild-type controls (*p* < 0.05) (Figure 4D). Consistent with behavioral improvements, ChAT-positive neuron number in NEP · 5XFAD as well as BACE1^+/−^ · NEP · 5XFAD mice was significantly higher than that of 5XFAD mice (*p* < 0.05) and equivalent to wild-type control levels. In contrast, the number of cholinergic neurons in BACE1^+/−^ · 5XFAD mice was as low as that of 5XFAD controls (*n* = 2), as reported in our previous work [15]. Together, these results indicate that neprilysin overexpression alone or in combination with BACE1 haploinsufficiency can prevent both contextual memory deficits and cholinergic neuron death in advanced stages of 5XFAD mice.

## Discussion

It is widely accepted that both accelerated Aβ production and compromised Aβ clearance underlie the molecular pathogenesis of AD [48,49]. In particular, the Aβ-generating enzyme BACE1 is increased [15,18,22,42,44,45,50,51] while the major Aβ-degrading enzyme neprilysin is decreased [18,42,52] during disease progression in 5XFAD mouse as well as human AD brains. Our previous studies using aged 5XFAD mice have shown that a monotherapeutic β-secretase-suppressing strategy is not sufficient to surmount robust Aβ accumulation, cholinergic neurodegeneration or cognitive deficits in advanced AD because of its failure to attenuate deleterious BACE1-elevating and neprilysin-reducing mechanisms [15,18,21]. In this study, we tested whether a combination of boosting neprilysin and inhibiting BACE1 activities may provide a useful approach to synergistically improve β-amyloidosis and memory deficits in 5XFAD mice already suffering from extensive plaque pathology. Consistent with previous results [15,16,18], BACE1 haploinsufficiency was no longer able to exert any beneficial effects in 5XFAD mice at 12 months of age. In contrast, transgenic overexpression of neprilysin in combination with BACE1 haploinsufficiency showed almost complete rescues of memory deficits of 5XFAD mice as assessed by the hippocampus-dependent contextual fear conditioning and their cholinergic neuron death in the medial septum. However, we also found that transgenic overexpression of neprilysin alone sufficed to exert similar beneficial or neuroprotective effects in aged 5XFAD mice. This can be accounted for by the findings that neprilysin overexpression was so robust (~8-fold higher than nontransgenic controls) that it almost completely prevented plaque development in the hippocampus and cerebral cortex of 5XFAD mice concomitant with significant reductions of total Aβ42 irrespective of the presence of BACE1^+/−^ gene deletion. Our observation is in agreement with previous work that showed greatly reduced plaque burden in hAPP-J20 mice crossbred with the same neprilysin transgenic line [29,30]. Collectively, it seems likely that strong Aβ-removing effects of overexpressed neprilysin in 5XFAD mice do not allow us to demonstrate the advantage of combining BACE1 reduction and neprilysin upregulation (if any) in further facilitating the beneficial outcomes.

Given that soluble Aβ oligomers may represent toxic mediators of synaptic and mnemonic failure in AD [53-55], we further compared changes in oligomeric forms of Aβ. Levels of Aβ oligomers were also significantly and equivalently reduced in NEP · 5XFAD and BACE1^+/−^ · NEP · 5XFAD mice, both of which showed memory improvements. Interestingly, we found that although BACE1^+/−^ · 5XFAD mice failed to exhibit significant mitigation of total Aβ accumulation or plaque pathology, similar levels of oligomer reductions occurred in brains of these mice, probably reflecting a consequence of lower BACE1-dependent Aβ production. Nevertheless, memory function remained deteriorated in BACE1^+/−^ · 5XFAD mice, suggesting that it is important to reduce both soluble Aβ oligomers and deposited insoluble Aβ species for the success of therapeutic interventions. This view is supported by accumulating evidence that indicates that Aβ plaques induce structural and functional disruption of neuron networks [56-60].

It should also be noted that there is some discrepancy concerning the relationship between neprilysin overexpression and improvements of hippocampus-dependent memory deficits in AD mouse models in spite of the consistent abolishment of plaque formation. Meilandt *et al*. [30] reported that transgenic overexpression of neprilysin under the αCaMKII promoter did not reduce pathogenic Aβ oligomers such as trimers and dodecamers (Aβ*56) in hAPP-J20 mice and thus failed to rescue spatial learning and memory deficits in the Morris water maze. In contrast, we showed that neprilysin overexpression in the same transgenic line significantly lowered Aβ oligomers and ameliorated contextual fear memory impairments in 5XFAD mice. Likewise, neprilysin overexpression under the PrP promoter in hAPP-J20 mice [61] or through the lentiviral vector-mediated gene transfer in APPswe/PS1dE9 mice [62] has been found to rescue spatial memory declines in the water maze, although Aβ oligomer levels were not measured in these studies. Conceivably, the controversial results may arise form different approaches used to overexpress neprilysin (e.g., the onset, amounts, neuronal populations of overexpression, etc.), AD mouse models applied and behavioral tasks. However, it is most likely that reductions of soluble Aβ oligomers in our NEP · 5XFAD model, despite the potential inability of neprilysin to directly degrade such Aβ assemblies [29], may be attributable to reduced *de novo* Aβ generation (via blocking BACE1-elevating mechanisms, as discussed below in more detail), leading to memory improvements in conjunction with the prevention of plaque deposition.

Although the degrees of Aβ reductions, mnemonic amelioration and neuronal protection were indistinguishable between NEP · 5XFAD and BACE1^+/−^ · NEP · 5XFAD mice, we found the advantage of combining BACE1 haploinsufficiency and neprilysin overexpression in more robustly suppressing the β-amyloidogenic processing of APP. Translational elevations of BACE1 expression (~2 folds relative to wild-type control levels) occurred in 12-month-old 5XFAD mice through overactivation of the eIF2α phosphorylation pathway, as reported previously [15,18,22,24,40,42]. Haploinsufficiency lowered BACE1 expression by ~50% in concordance with the reduction of gene copy number, while eIF2α phosphorylation was not affected and consequently BACE1 remained upregulated (i.e., equivalent to wild-type levels in spite of the ablation of a single BACE1 allele) in BACE1^+/−^ · 5XFAD mice. Interestingly, we found that translational elevation of BACE1 was completely blocked concomitant with a significant reduction of eIF2α phosphorylation in NEP · 5XFAD mice. This observation is consistent with our recent evidence directly demonstrating that genetic inhibition of the PERK-mediated eIF2α phosphorylation pathway can reverse BACE1 upregulation in 5XFAD mice [42,43]. It has been proposed that Aβ accumulation induces BACE1 elevation in neurons in the close vicinity of plaques, which in turn further accelerates Aβ generation and plaque growth in 5XFAD mouse as well as human AD brains [22-26]. Therefore, it is conceivable that Aβ-degrading effects of overexpressed neprilysin prevent Aβ accumulation in 5XFAD brains, thereby suppressing the eIF2α phosphorylation-dependent upregulation of BACE1 expression associated with plaques. More importantly, we demonstrate that neprilysin overexpression in combination with BACE1 haploinsufficiency in 5XFAD mice almost completely abolishes eIF2α phosphorylation, resulting in BACE1 expression below wild-type controls in BACE1^+/−^ · NEP · 5XFAD mice as expected by a single BACE1 allele ablation under the reversal of translational upregulation.

## Conclusions

The present study shows that transgenic overexpression of neprilysin is sufficient to reduce Aβ accumulation and almost completely prevent cholinergic neuron loss and memory deficits in aged 5XFAD mice. Accordingly, there is no significant difference in AD-like phenotypes between NEP · 5XFAD and BACE1^+/−^ · NEP · 5XFAD mice. In the experimental setting with robust neprilysin overexpression (~8-fold), it seems difficult to unequivocally demonstrate the advantage of combining partial BACE1 inhibition and neprilysin upregulation in further facilitating their beneficial outcomes during advanced stages of AD. However, the results presented here provide clear evidence that boosting neprilysin-dependent degradation of Aβ can block BACE1-elevating signaling mechanisms associated with progressive plaque formation in 5XFAD mice. Consequently, BACE1 expression and the direct β-metabolite of APP (C99) in BACE1^+/−^ · NEP · 5XFAD mice are further reduced to the levels reflecting a combination of single BACE1 allele ablation (BACE1^+/−^) and the abolishment of translational BACE1 upregulation (NEP). These findings suggest that activation of neprilysin function may be useful for complementing the limited efficacy of direct BACE1 suppression in reducing β-amyloidogenic processing of APP in advanced AD. This is quite important given that over-inhibition of β-secretase activities is suggested to induce potentially mechanism-based adverse effects on the basis of an increased number of novel BACE1 substrates that have been discovered beside APP [4,5,63,64]. Our gene-based findings warrant further mechanistic investigations of synergistic benefits that may be brought about by combined treatments with β-secretase inhibitors (e.g., GRL-8234 and TAK-070) [19-21] and feasible therapeutic agents capable of moderately increasing neprilysin expression or activities (e.g., γ-hydroxybutyrate) [65] in animal models relevant to later phases of AD with established Aβ pathology.

## Methods

### Subjects

We used 5XFAD mice (Tg6799 line) that co-overexpress FAD mutant forms of human APP (Swedish mutation: K670N, M671L; Florida mutation: I716V; London mutation: V717I) and PS1 (M146L and L286V mutations) transgenes under transcriptional control of the neuron-specific Thy-1 promoter [9,10,31]. Hemizygous 5XFAD transgenic mice (C57Bl/6 background) were bred to heterozygous BACE1 knockout (BACE1^+/−^) mice (C57Bl/6 background) (Stock number: 004714, The Jackson Laboratory, Bar Harbor, ME, USA) [11,66] to obtain bigenic mice with the BACE1^+/−^ · 5XFAD^+/−^ genotype. BACE1^+/−^ · 5XFAD^+/−^ mice were further crossbred to hemizygous neprilysin transgenic mice (C57Bl/6 background) (Stock number: 005086, The Jackson Laboratory) that overexpress human neprilysin under control of the αCaMKII promoter [29,30], yielding animals with the genotypes of interest. Genotyping was performed by PCR analysis of tail DNA. All experiments were done blind with respect to the genotype of mice at 12 months of age when 5XFAD control mice showed extensive Aβ plaque pathology that was accompanied by significant BACE1 elevation and neprilysin reduction and no longer responsive to mitigation by partial BACE1 suppression [15,16,18,21]. Procedures were conducted in accordance with the National Institutes of Health *Guide for the Care and Use of Laboratory Animals* and approved by the Nathan Kline Institute Animal Care and Use Committee.

### Contextual fear conditioning

Contextual fear conditioning was tested as described previously [14,32,67]. In this behavioral assay, mice learn to associate a distinct context (CS: conditioned stimulus) with aversive footshock (US: unconditioned stimulus) through hippocampus-dependent mechanisms [68,69]. During training, mice were placed in the conditioning chamber for 3 min and then received a footshock (0.8 mA, 2 s). After the shock delivery, mice were left in the chamber for another 30 s. Contextual fear memory was evaluated by scoring freezing behavior (the absence of all movement except for that needed for breathing) for 3 min when the mice were placed back into the same conditioning chamber 24 h after training. The automated FreezeFrame system (Coulbourn Instruments, Allentown, PA, USA) was used to score the amount of freezing. After behavioral testing, some mice were sacrificed for immunoblotting and ELISA experiments, and others were perfused for immunohistochemistry.

### Immunoblot analysis

Hemibrain samples were taken from the mice under deep isoflurane anesthesia and were snap-frozen for biochemical assays. For western blot analysis, each sample was homogenized in 8-fold volumes of cold homogenization medium containing 70 mM sucrose, 210 mM mannitol, 2 mM HEPES, 0.1 mM EDTA and protease/phosphatase inhibitor cocktail and centrifuged at 10,000 g for 10 min to remove any insoluble material. Protein concentrations were determined by a BCA protein assay kit (Pierce, Rockford, IL, USA), and 10–50 μg of protein was run on NuPAGE 4–12% or 10% Bis-Tris gels (Invitrogen, Carlsbad, CA, USA) and transferred to nitrocellulose membrane. After blocking, membranes were probed with the following primary antibodies: anti-neprilysin (1:1,000, ab951, Abcam, Cambridge, MA, USA), anti-BACE1 (1:1,000, B0681, Sigma-Aldrich, St. Louis, MO, USA), an antibody that recognizes C-terminal epitope in APP (1:1,000, C1/6.1, kindly provided by Dr. Paul Mathews, Nathan Kline Institute) to detect full-length APP/C-terminal fragments, anti-phospho-eIF2α (Ser51) (1:1,000, #3398, Cell Signaling Technology, Danvers, MA, USA), anti-eIF2α (1:2,000, #9722, Cell Signaling Technology), and anti-β-actin (1:15,000, AC-15, Sigma-Aldrich). They were then incubated with horseradish peroxidase-conjugated secondary IgG. Immunoblot signals were visualized by an ECL chemiluminescence substrate reagent kit (Pierce) and quantified by densitometric scanning and image analysis using Quantity One software (Bio-Rad Laboratories, Hercules, CA, USA). Duplicates of each sample in immunoblot assays were averaged for comparison between groups.

### ELISAs of soluble Aβ oligomers and total Aβ42

To measure the concentrations of soluble Aβ oligomers, each hemibrain sample was homogenized in 8-fold volumes of homogenization medium, as described above. To quantitate total levels of Aβ42, the other hemibrain was extracted in 8-fold volumes of cold 5 M guanidine HCl plus 50 mM Tris HCl (pH 8.0) buffer and centrifuged at 20,000 g for 1 h at 4°C to remove insoluble material. Final guanidine HCl concentrations were below 0.1 M. Protein concentrations were determined by a BCA protein assay kit (Pierce). Supernatant fractions were analyzed by well-established human Aβ ELISA kits specific to oligomeric forms of Aβ (27725, IBL America, Minneapolis, MN, USA) and Aβ42 (KHB3441, Invitrogen) according to the protocols of the manufacturers. Optical densities at 450 nm of each well were read on a VersaMax tunable microplate reader (Molecular Devices, Sunnyvale, CA, USA), and sample Aβ oligomer and Aβ42 concentrations were determined by comparison with the respective standard curves. Aβ oligomer and Aβ42 concentration values were normalized to total brain protein concentrations and expressed in picograms and nanograms per milligram of total protein, respectively.

### Aβ immunohistochemistry

Mice were transcardially perfused with 0.1 M phosphate buffered saline (PBS, pH7.4), followed by 4% paraformaldehyde in PBS under deep isoflurane anesthesia. Brains were postfixed for 24 h in 4% paraformaldehyde in PBS at 4°C and transferred to PBS. The brain was sectioned coronally at 30 μm using a vibratome (VT1200, Leica Microsystems, Wetzlar, Germany), and successive sections were stored in PBS containing 0.05% sodium azide at 4°C. Three sections per mouse taken at levels between −1.7 and −1.9 mm to bregma according to the mouse brain atlas of Franklin and Paxinos [70] were stained by the avidin-biotin peroxidase complex (ABC) method as described previously [13,14,71]. Briefly, the sections were incubated overnight at 4°C with mouse monoclonal anti-Aβ1–16 (6E10) antibody (1:200, SIG-39347; Covance, Princeton, NJ, USA). The ABC kit (PK-2200; Vector Laboratories, Burlingame, CA, USA) was utilized with 3,3’-diaminobenzidine tetrahydrochloride (DAB) as a chromogen to visualize the reaction product. The sections were then mounted on charged slides, dehydrated in a series of alcohol, cleared in xylene and covered with a coverslip. Light microscopy was conducted on an Axioskop 2 microscope equipped with an AxioCaM HRc digital camera (Zeiss, Oberkochen, Germany) for capturing images. Semi-quantitative analysis was performed using AxioVision imaging software with the AutoMeasure module (Zeiss). The threshold optical density that discriminated staining from background was determined and held constant for all quantifications. Identified objects were individually inspected by the same investigator to confirm the object as a plaque or not in a blinded manner. Percentage area occupied by Aβ deposits in the hippocampus and cortex was assessed bilaterally to compare plaque burdens between groups.

### ChAT immunohistochemistry

Three brain sections per mouse were stained by the ABC method for immunohistochemical analysis of ChAT-positive neurons in the Ch1/2 comprising the medial septum and the vertical limb of the diagonal band, as described [15]. The sections were taken at levels between +1.2 and +0.8 mm to bregma according to the atlas of Franklin and Paxinos [70] and incubated overnight at 4°C with polyclonal goat anti-ChAT antibody (1:200; AB144P, Millipore, Billerica, MA, USA). The DAB staining was performed using the ABC kit (PK-6105, Vector Laboratories). After identified objects, following thresholding under an Axioskop 2 microscope (Zeiss), were individually inspected in a blinded manner to confirm the object as a neuron or not, the number of ChAT-positive neurons in the Ch1/2 was counted using AxioVision imaging software (Zeiss). The average of ChAT-positive neuron number per section from each mouse was used to calculate group medians.

### Statistical analysis

Significant differences between the groups were determined by a one-way ANOVA and *post-hoc* Fisher’s PLSD tests were applied following all ANOVAs showing significance. Data were presented as mean ± SEM and the level of significance was set for *p* value less than 0.05.
